# Multidimensional fluorescence microscopy of multiple organelles in Arabidopsis seedlings

**DOI:** 10.1186/1746-4811-4-9

**Published:** 2008-05-19

**Authors:** Naohiro Kato, Dexter Reynolds, Matthew L Brown, Marietta Boisdore, Yukichi Fujikawa, Andrea Morales, Lee A Meisel

**Affiliations:** 1 Louisiana State University, Department of Biological Sciences, Baton Rouge, LA, 70803, USA; 2 Southern University and A&M College, Baton Rouge, LA, 70813, USA; 3 Millennium Nucleus in Plant Cell Biology and Center of Plant Biotechnology, Andres Bello University, Av. República 217, 837-0146 Santiago, Chile

## Abstract

**Background:**

The isolation of green fluorescent protein (GFP) and the development of spectral variants over the past decade have begun to reveal the dynamic nature of protein trafficking and organelle motility. *In planta *analyses of this dynamic process have typically been limited to only two organelles or proteins at a time in only a few cell types.

**Results:**

We generated a transgenic Arabidopsis plant that contains four spectrally different fluorescent proteins. Nuclei, plastids, mitochondria and plasma membranes were genetically tagged with cyan, red, yellow and green fluorescent proteins, respectively. In addition, methods to track nuclei, mitochondria and chloroplasts and quantify the interaction between these organelles at a submicron resolution were developed. These analyzes revealed that N-ethylmaleimide disrupts nuclear-mitochondrial but not nuclear-plastids interactions in root epidermal cells of live Arabidopsis seedlings.

**Conclusion:**

We developed a tool and associated methods for analyzing the complex dynamic of organelle-organelle interactions in real time *in planta*. Homozygous transgenic Arabidopsis (Kaleidocell) is available through Arabidopsis Biological Resource Center.

## Background

Advances in fluorescence microscopy [1] and the development of multiple spectral variants of the green fluorescent protein (GFP) [2] have revolutionized plant cell biology. These technological developments have produced a wealth of published information related to protein localization, and are currently being exploited to analyze protein-protein interactions [3]. Recently, this fluorescence technology has begun to be applied to an analysis of the dynamic motility of several organelles, including mitochondria [4-6] nuclei [7] and Golgi [8]. Much of this information has been deposited in the plant organelle database (PODB) [9]. However, the majority of these plant studies have been performed using transient assays or have involved marking only a few organelles with one or two fluorescent proteins, making it difficult to perform detailed analyses of the interaction or association between multiple organelles. In both plants and animals, some organelles are localized in close proximity to one another such that the distance that separates them is shorter than the resolution limit of a conventional fluorescence microscope (< 300 nm), but longer than the resolution limit of a conventional electron microscope (> 20 nm). In the context of the studies presented here, we describe these close localizations of organelles as organelle-organelle interactions (OOIs).

A classical example of OOIs in plants are those involving chloroplasts, peroxisomes and mitochondria in green tobacco leaves, observed by electron microscopy [10]. Because enzymes required for photorespiration are stored separately in these organelles, secondary metabolites, such as glycolate and glycine, must be transported among these organelles. Hence, in this case, the OOIs may facilitate photorespiration by maintaining short transportation distances. Using confocal microscopy, physical interactions between the nucleus and plastids were recently described in tobacco hypocotyl epidermal cells [11], and signal transduction between the nucleus and plastids has also been reported [12]. Kwok and Hanson have hypothesized that nucleus-plastid signaling may be facilitated by OOIs that reduce the distance between the nucleus and plastids [11]. In animal cells, OOIs between the nucleus and mitochondria have also been described [13]. In a study of ATP transport, physical interactions between nuclei and mitochondria were observed in cultured cells using both confocal and electron microscopy. Although nuclei are not capable of producing ATP, a large amount of it is required for nucleus-specific functions, such as transcription and mRNA transport. Thus, nucleus-mitochondria OOIs may facilitate ATP transport from the mitochondria to the nucleus [13].

Although biologically important roles for OOIs have been hypothesized as described above, the nature of organelle-organelle physical interactions is poorly understood. Indeed, molecules that mediate the physical interactions between nuclei and plastids or mitochondria have yet to be identified. Organelles may interact with each other through an active molecular recognition mechanism or perhaps, if cellular space is limited, through a passive process. In this study, we sought to determine which phenomenon – active or passive recognition – is correct by addressing the following questions:

1. How many plastids and mitochondria are in each cell?

2. How much space is available in a cell for the nucleus, plastids and mitochondria?

3. Where do these organelles locate in the three-dimensional (3-D) space of the cell?

4. How long do the OOIs last?

5. Are specific molecular interactions involved in mediating OOIs?

To begin to answer these questions, we have developed a transgenic Arabidopsis plant as a tool for analyzing the dynamics of multiple organelles and multiple OOIs in real time *in planta*.

## Results and Discussion

### Establishing homozygous Kaleidocell lines

**Figure 1:**
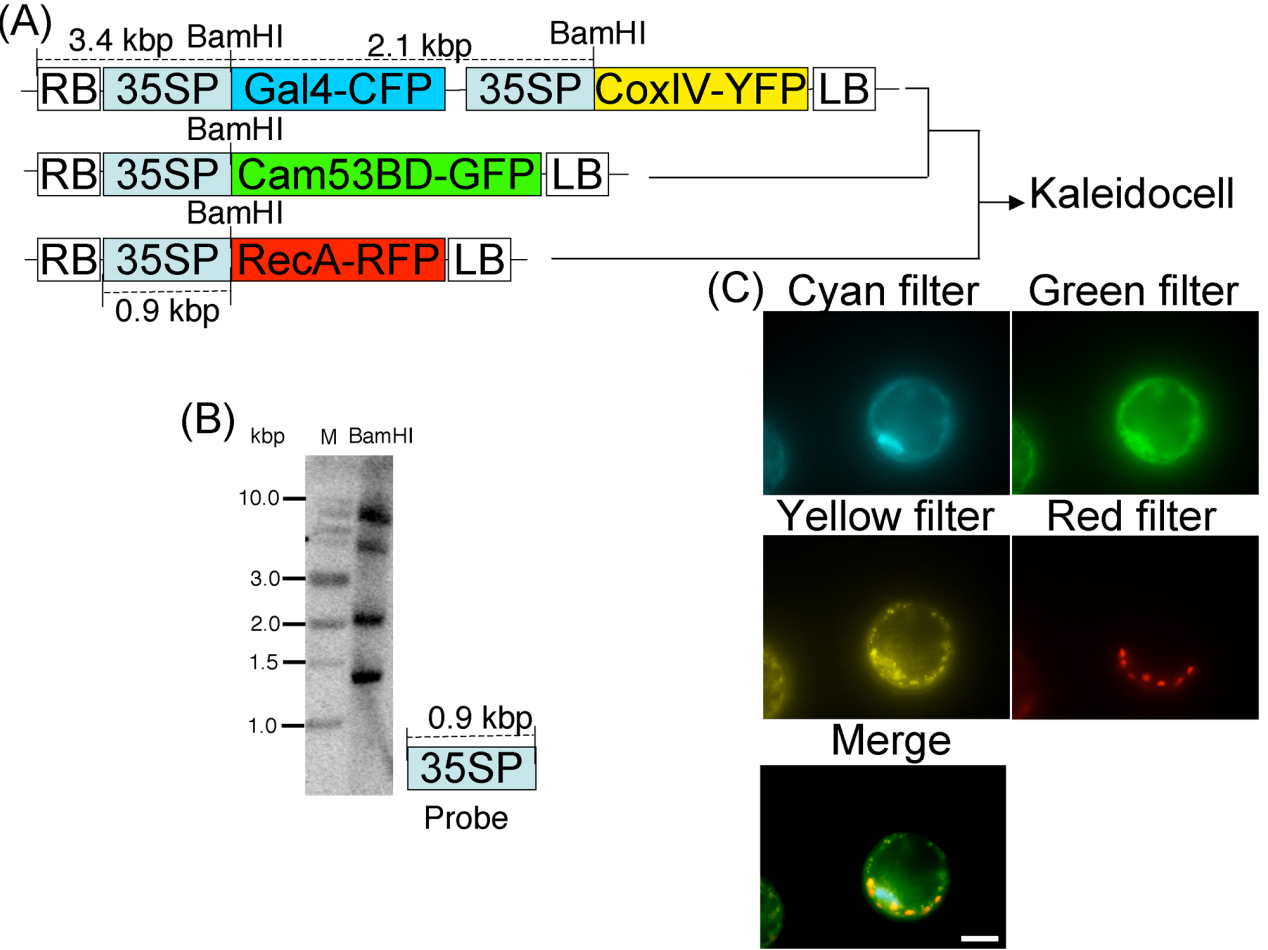
**Transgene constructs and their expression in the Kaleidocell line**. **(A) **The fusion genes were inserted downstream of the 35S cauliflower mosaic virus promoter (35SP). Expression cassettes of Gal4-CFP and CoxIV-YFP were inserted in tandem in the transgene region [between the right (RB) and left borders (LB)]. Expression cassettes of Cam53BD-GFP and RecA-RFP were inserted in the transgene region. Unique sites digested by B*am*HI in the transgenes and the predicted sizes (kbp: kilo base pairs) between the B*am*HI sites and to the RB were shown on the top of the constructs. The size of the 35SP is also shown on the bottom of the construct. A transgenic plant expressing Gal4-CFP and CoxIV-YFP was crossed with a transgenic plant expressing Cam53BD-GFP. The resulting plant was further crossed with a transgenic plant expressing RecA-RFP. The final transgenic plant expressing all four transgenes was designated as the Kaleidocell line. Gal4: *Saccharomyces cerevisiae *nuclear protein, CoxIV: *Saccharomyces cerevisiae *cytochrome oxidase IV, Cam53BD: petunia calmodulin CaM53 binding domain, RecA: *Arabidopsis *DNA repair protein. **(B) **Southern blotting analysis of the B*am*HI digested genomic DNA of homozygous Kaleidocell. Lane M: 1 kb DNA Ladder (NEB), Lane BamHI: B*am*HI digested genomic DNA. The sizes of the DNA ladder are shown on the left. The DNA probe is shown on the right bottom. **(C) **Fluorescence microscope images of a protoplast from the Kaleidocell line. The protoplast was observed with four different fluorescence filter sets: cyan, green, yellow, or red. Captured images were merged to generate a single image of the protoplast. Scale bars = 10 μm.

Transgenic *Arabidopsis *plants, designated Kaleidocell (*in Greek, kalos *beautiful + *eidos *form + cell), were engineered to contain multiple fluorescent proteins that label organelles *in vivo*. Specifically, nuclei, plastids, mitochondria and plasma membranes were genetically tagged with cyan fluorescent protein (CFP), red fluorescent protein (RFP), yellow fluorescent protein (YFP) and green fluorescent protein (GFP), respectively (Figure 1A). The fluorescent proteins and tagged peptides used in this study have been shown previously to be sufficient to direct fusion proteins to each cellular component in plants. Nuclei were tagged with ECFP (Clontech, CA) fused to the yeast nuclear localized protein, Gal4 [14], and the nuclear localization signal from SV40 large T-antigen [15]. Plastids were tagged with DsRed2 (Clontech) fused to the Arabidopsis RecA signal peptide [16]. Mitochondria were tagged with EYFP (Clontech) fused to the mitochondrial localization signal sequence in yeast cytochrome oxidase IV [17]. Plasma membranes were tagged with sGFP [18] fused to the petunia calmodulin CaM53 [19,20].

To confirm transgene integration and to predict transgene copy numbers, we performed Southern blots on BamHI-digested genomic DNA from homozygous Kaleidocell using a probe encoding the promoter region of each of these transgenes (Figure 1B). We detected four bands, indicating that a single copy of each expression cassette (CFP, RFP, YFP, and GFP) was integrated in the Kaleidocell genome. One of the bands appeared at approximately 1.4 kbp, which is 2.0 kbp shorter than expected-shortest size (3.4 kbp). The reduced size of this band suggests that one of the T-DNAs did not integrate completely into the genome. However, the fact that all four spectral variants were visible in Kaleidocell specimens indicates that this truncation of T-DNA had no apparent effect on the functionality of the four transgenes.

To confirm proper accumulation of each fluorescent protein, protoplasts were generated from one-week-old homozygous Kaleidocell seedlings (Figure 1C). Within a single protoplast, we detected cyan, red, yellow, and green fluorescence in the nucleus, plastids, mitochondria, and plasma membrane, respectively. The fluorescence spectrums of CFP and GFP overlap with one another, as do those of GFP and YFP. Accordingly, it can be challenging to distinguish CFP, GFP and YFP signals in a single cell [21]. Although these signals can be separated through the use of spectral imaging and linear un-mixing of image data [22], the microscopes and software used in this study did not allow us to do so. Furthermore, the petunia calmodulin CaM53 that tags plasma membranes tended to accumulate in nuclei by blocking isoprenoid biosynthesis or depleting carbons [19]. To distinguish organelles from one another in this study, we used a combination of fluorescent signals and organelle size. The nucleus was a clearly recognizable entity with a diameter larger than 5 μm. Meanwhile, plastids were entities with diameters just larger than 2.5 μm, and mitochondria were the smallest recognizable objects with a diameter greater than 0.7 μm.

**Figure 2:**
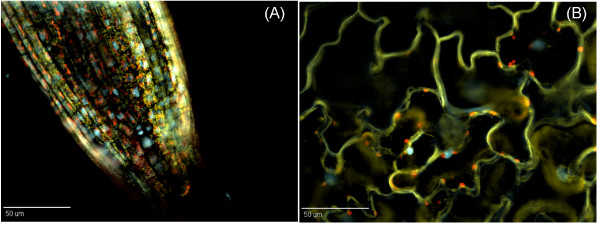
**Expression of the transgenes in seedlings in the Kaleidocell line**. **(A) **Fluorescence micrograph of a root meristem from the 7 days-old Kaleidocell line. The root meristem was observed with three different fluorescence filter sets: cyan, yellow, and red. Captured images were merged to generate a single image. **(B) **Fluorescence micrograph of epidermal cells from the cotyledon of a 7 day-old Kaleidocell plant. The cotyledon was observed with three different fluorescence filter sets: cyan, yellow, and red. Captured images were merged to generate a single image. Scale bars = 50 μm.

The fluorescent proteins in Kaleidocell seedlings (3 days to 2 weeks post-germination) were easily detected in the hypocotyls and roots of all individuals analyzed (Figure 2A and 2B). However, three weeks post-germination, the fluorescent protein signals were not as readily detected. Although the reason for this time-dependent reduction in signal intensity is unknown and requires further study, it is likely that the transgene loci may be affected by local chromatin modifications, which alter transgene expression levels at distinct stages of plant development.

Because Kaleidocell is homozygous for all four fluorescent proteins, plants can be propagated without using a selection reagent such as kanamycin. Additionally, we have found that the fluorescence of all four fluorescent proteins remains stable in subsequent generations (data not shown).

### Mounting living seedlings on a microscope stage

**Figure 3:**
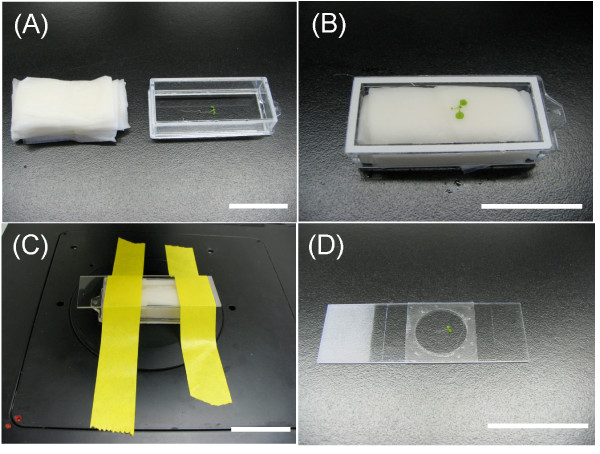
**Mounting a seedling on an inverted fluorescence microscope**. **(A) **Preparation: each seedling was laid on a chamber-coverglass, and layers of Kimwipes were folded and soaked with distilled water. **(B) **Mounting: water-soaked Kimwipes were wrung out onto the chamber-coverglass. A bottom view of the chamber-coverglass is shown. **(C) **Microscope stage setting: a microscope slideglass was used as a lid for the chamber-glass. The chamber-glass was fixed on the stage with labeling tape. **(D) **Alternative mounting method. Scale bars = 4 cm.

Living plants can be adhered to a coverglass for a number of hours using a gelatin- or agar-based gel mounting medium [23]. Although this method generally works well, it is not suitable for high-resolution 3-D microscopy because of optical scattering and aberrations caused by the gel (Kato unpublished). To optimize the quality of the signal captured from the Kaleidocell plant, we developed a simple method that allowed us to make extended observations (hours) of whole seedlings with minimum optical scattering or other aberrations. Our method uses water-soaked Kimwipes to provide 1) a mounting medium (water) that fills the empty space between the sample and the coverglass and reduces dehydration of the sample being analyzed, and 2) a force that gently pushes the seedling towards the coverglass (Figure 3A–C). During our experiments, *Arabidopsis *seedlings mounted in this manner grew for over 12 hours on a microscope stage (data not shown).

In addition, a convenient method also was developed to enclose Kaleidocell in a microscope slide (Figure 3D). After germinating seeds for 3 days on water-soaked blotting paper in a plastic Petri dish, a microscope slide with a one-well adhesive spacer (20 mm in diameter, 0.12 mm deep) was used to mount Kaleidocell with water. A glass cover slip was used to seal a seedling in this chamber. Although it may be difficult to continuously observe single cells for extended periods due to the gradual movement of Kaleidocell in water, it is possible to obtain sequential high-resolution images over a short period of time (e.g., 10 minutes). This method may be useful to quickly test or demonstrate the feasibility of Kaleidocell.

### Detection of nuclei, plastids and mitochondria in epidermal and cortical cells of the Kaleidocell root

**Figure 4:**
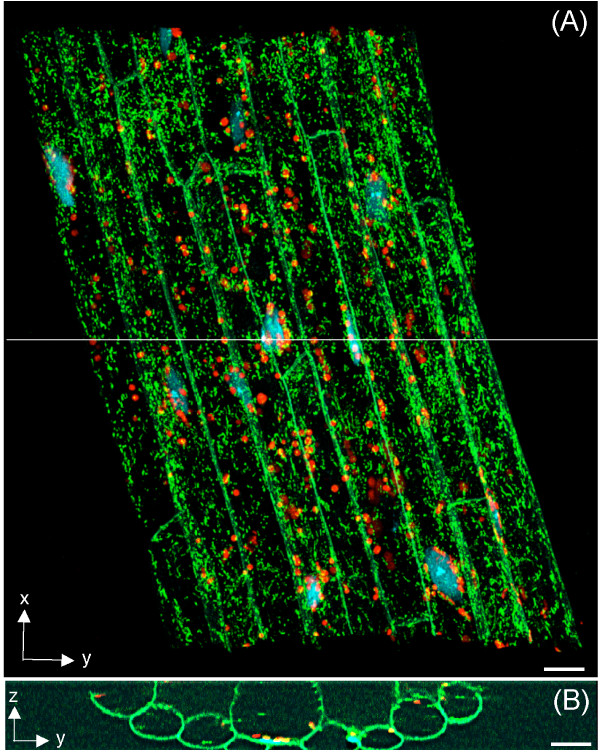
**Detection of nuclei, plastids, and mitochondria in a Kaleidocell root**. **(A) **A maximum projection image of a vertical series of scanning confocal micrographs of a root of a Kaleidocell seedling. The root grew along the x-axis. A total of 17 epidermal and 8 cortical cells (green lines) were identified. Cell boarders at the ends of the x-axis (top and bottom of the image) were beyond the scanning area. Within a total of the 25 cells, 11 nuclei (blue spots), 255 plastids (red spots), and 10,158 mitochondria (green spots) were identified. A white thin horizontal line in the middle of the image indicates a point where a cut-through image was generated. **(B) **Cut-through image of the stacked image. Image contrast was adjusted to enable clear visualization of the cell borders. Scale bars = 10 μm.

To visualize the multiple fluorescent proteins marking the different organelles in Kaleidocell, we obtained a stacked confocal scanning microscopic image of a Kaleidocell seedling root (Figure 4). The resolution of the objective lens used in this scan was calculated at 0.25 μm in the x- and y-axes and 0.65 μm in the z-axis. Because of the limitations of our laser lines, we acquired GFP (plasma membranes) and YFP (mitochondria) signals in the same channel. However, as noted above, the size and pattern of each of these organelles made it easy to distinguish the plasma membrane from the mitochondria.

A total of 25 cells, 17 epidermal and eight cortical, were clearly identified within a 230.2 × 230.3 × 30.9 μm scan area (Figure 4). Of the 25 identified cells, 11 nuclei were inside the scan area during acquisition. The number of plastids and mitochondria were determined using imaging software. Point-like structures (spots) were automatically identified, and 255 RFP spots (diameter > 2.5 μm) and 10,158 GFP/YFP spots (diameter > 0.7 μm) were detected. Hence, we estimated that a single cell in the root of the Kaleidocell seedling contained approximately 10 plastids and 400 mitochondria. Mitochondria do not exist in cells as discrete units and could be smaller than our microscopic resolution [4,5], so the actual number of mitochondria may be higher or lower than our reported numbers. However, the number suggested by our analysis is similar to that predicted by the amount of mitochondrial DNA in animal cells [24], providing a measure of confidence that the number of mitochondria we were able to visualize in a single cell would be close to the actual number.

### Three-dimensional positioning of nuclei, plastids and mitochondria

**Figure 5:**
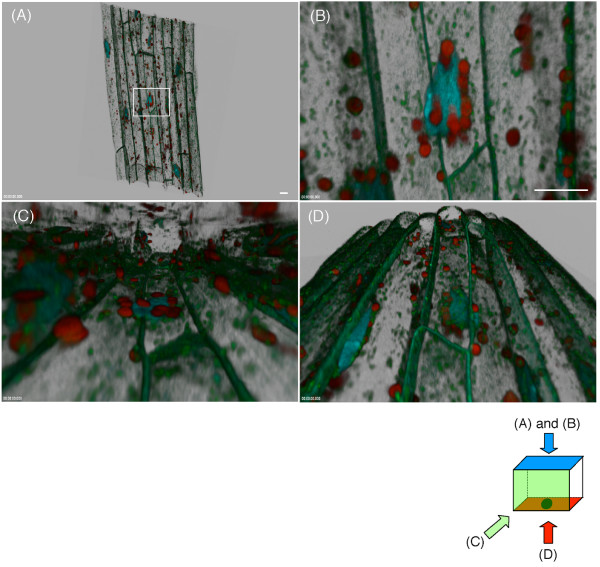
**Localization of nuclei, plastids, and mitochondria in a 3-D model**. The stacked image was converted to a 3-D model (blends projection). In this projection, viewing directions and their transparencies were blended to produce a 3-D perspective of the structure. Viewpoints of each image (A to D) are indicated on the bottom. The box indicates the 3-D coordination of the stacked image. The different colors on each wall indicate the different dimensions. Arrows with a letter indicate the viewpoints of each image. A black ellipse on the bottom of the box indicates the position of a nucleus. **(A) **The blends projection of the stacked image of Figure 4(A). A white rectangle indicates the area enlarged in (B, C, and D). **(B) **Top view. A nucleus (blue) interacts with plastids (red) and mitochondria (green). The 3-D coordination was not clear in this viewpoint. **(C) **Side view of the same nucleus. Note that the plastids lay on the nucleus. Also note that the majority of the organelles localize to the cortex of the cells. **(D) **Bottom view of the same nucleus. Note that the nucleus lays on the mitochondria. Scale bars = 10 μm.

The stacked confocal scanning microscopic image was converted to a 3-D model and visually inspected using imaging software to determine the location of organelles in space (Figure 5 and see Additional file 1). We found that almost all organelles were located in the cortical region of the cells. By manually identifying cellular locations of nuclei, plastids and mitochondria, we estimated that 60 ± 12.5% (n = 10) of the cell volume did not contain these organelles. This suggests that the cytosolic area in which nuclei, plastids and mitochondria can move comprises approximately 40% of the total cellular volume in epidermal and cortical cells of *Arabidopsis *roots. However, it has been demonstrated previously that expanded vacuoles in root cells [25] push nuclei, plastids and mitochondria to the cortex. Thus, the total cellular volume available to these organelles may vary as a function of vacuole size and/or complexity in different cells or cell types.

### Dynamics of nuclear interactions with plastids and mitochondria

**Figure 6:**
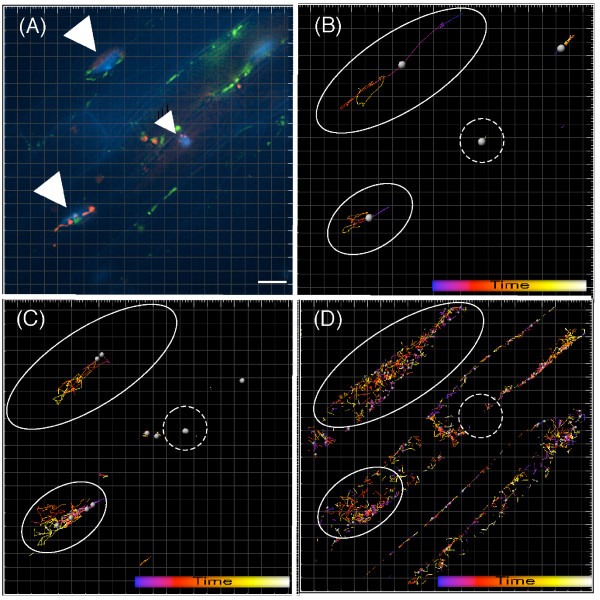
**Movements of nuclei, plastids, and mitochondria**. Stacked deconvolution micrographs of root epidermal cells during a 3 h and 40 min long time-lapse observation. **(A) **Image captured at 2 h and 46 min into the observation period. Nuclei, plastids, and mitochondria appear blue, red, and green, respectively. Triangles indicate the nuclei. Scale bar = 10 μm. **(B) **Movements of nuclei tracked from 2 h and 27 min to 3 h and 40 min (a total tracking duration of 103 min). Each track is displayed as a line. Line color indicates the time point, corresponding to the time color bar on the right bottom. Solid-line circles indicate areas where the nuclei moved. Dashed-line circles indicate an area where the nucleus stalled. **(C) **Movements of plastids. Movements are indicated by solid- and dashed-line circles as in (B). **(D) **Movements of mitochondria. Movements are indicated by solid- and dashed-line circles as in (B). Notice that the tracks of the plastids and mitochondria are similar to that of the nuclei.

To simultaneously investigate the dynamics of OOIs between nuclei and plastids, and nuclei and mitochondria, we tracked the movements of each organelle in a single root epidermis for 3 h, at 1 min intervals (Figure 6, Table 1, and see Additional file 2).

**Table 1:** Tracking analysis of nuclei, plastids and mitochondria at 1 min intervals for 103-min duration.

Tracking	Nuclei	Plastids	Mitochondria
Numbers	4	128	805
Duration (min)	8 – 70	1 – 70	1 – 34
Length (μm)	4 – 122	0 – 88	0 – 44
Speed (μm/min)	0 – 2.4	0 – 4.2	0 – 112.8

During this time, we observed nuclear movements similar to those reported previously [7]. For example, one nucleus moved constantly at an average speed of 2.4 μm/min for 70 min covering a distance of 122 μm; another nucleus within the same tissue did not move as far (4-μm travel distance over 70 min, Figure 6 and see Additional file 3). A previous study indicated that nuclear movements in *Arabidopsis *root hairs correlated with cell growth [26]. Hence, the growth status of individual cells in the root epidermis may be reflected in nuclear movements.

Plastid movements have also been previously reported to vary [6]. We found the plastids that interacted with nuclei moved with the nuclei over the 3-h observation interval (Figure 6 and see Additional file 4), indicating that the nucleus constantly interacts with the same population of plastids, independent of their movement within the cell.

The mitochondria were smaller and moved faster than the other organelles, making them difficult to track individually. The software we used was unable to track the movement of the mitochondria for an extended period (Table 1). However, we did observe that the mitochondria that interacted with and surrounded the nuclei moved in the same direction as the nuclei (Figure 6 and see Additional file 5). Although this does not conclusively demonstrate that the nucleus was interacting with the same population of mitochondria, it does suggest that the nucleus is constantly interacting with mitochondria.

### Pharmacological analysis of OOIs between nuclei and plastids or mitochondria

Unlike most other eukaryotes, actin microfilaments control organelle movements in higher plants (reviewed in [27,28]). Actin microfilaments, together with myosin motor proteins, also control cytoplasmic streaming [27]. It is also known that microtubules, which control organelle movements in other eukaryotes, contribute to the positioning of organelles in higher plants[28].

To explore the nature of the nucleus-plastid and nucleus-mitochondria OOIs, we performed a pharmacological screen to search for factors that might alter these OOIs. We exposed Kaleidocell seedlings to five different pharmacological compounds that have been used previously to study the positions and movements of nuclei, mitochondria or plastids in higher plants [7,29-31]. The first compound, Latrunculin B, decreases the amount of polymerized actin and immobilizes nuclei [7] and mitochondria [29]; it also disrupts plastid positioning [30]. The second compound, oryzalin, influences tubulin polymerization and disrupts the positioning of mitochondria [29]. The third compound, 2,4-dinitrophenol (DNP), accelerates depletion of ATP from the cells and immobilizes mitochondria [29]. The fourth compound, 2-monoxime (BDM), is a mammalian myosin ATPase inhibitor that immobilizes mitochondria and also causes immobilized mitochondria to aggregate [29]. The fifth compound, N-ethylmaleimide (NEM), causes alkylation of proteins and immobilizes nuclei [7], mitochondria [29] and plastids [31], and also causes immobilized mitochondria to aggregate [29].

**Figure 7:**
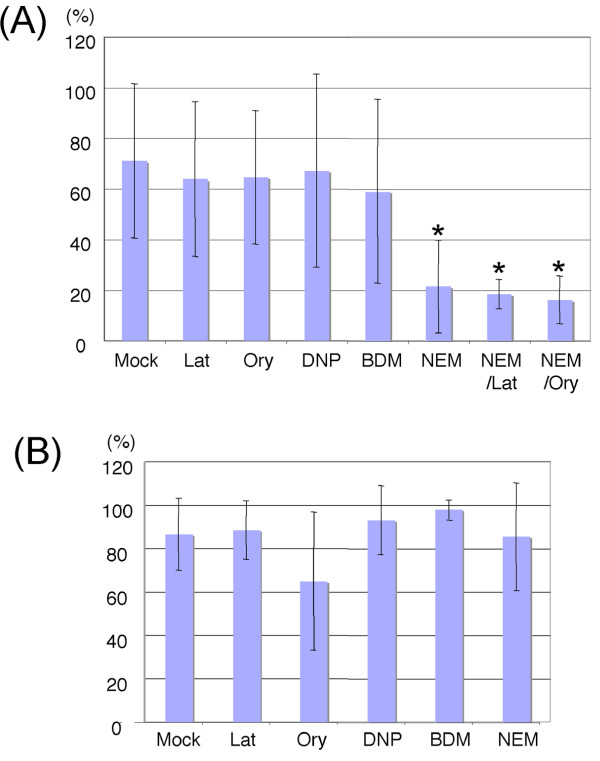
**Effects of pharmacological compounds on nucleus-mitochondria and nucleus-plastid interactions**. Rates of the nuclei interacting with mitochondria (A) or plastids (B) in the root epidermal cells were scored. The columns show averaged interaction-rates in the observed cells with standard deviations. Significantly different (P ≤ 0.01) rates from the mock treated samples are indicated with asterisks. Mock: 0.1% (v/v) dimethyl sulfoxide (N = 128), Lat: 40 μM latrunculin B (N = 88), Ory: 10 μM oryzalin (N = 86), DNP: 40 μM 2, 4-dinitrophenol (N = 34), BDM: 20 μM 2, 3-butanedione monoxime (N = 42), NEM: 50 μM N-ethylmaleimide (N = 84), NEM/Lat: 50 μM N-ethylmaleimide and 40 μM latrunculin (N = 61), NEM/Ory: 50 μM N-ethylmaleimide and 10 μM oryzalin (N = 62).

Nucleus-mitochondria interactions and nucleus-plastid interactions were manually identified under a fluorescence microscope using size, morphology and spectral fluorescence to identify each type of organelle (Figure 7). In the absence of pharmacological compound exposure, 71% of nuclei were observed to interact with mitochondria (n = 128, 6 independent experiments). This percentage was lower than that obtained using confocal imaging (~100% of the observed nuclei), suggesting that the lower resolution of the objective lens used on the epifluorescence microscope may limit the ability to identify all nucleus-mitochondria OOIs. Alternatively, the inability to acquire 3-D images may have been an issue, resulting in mitochondria located beneath nuclei being missed during OOI detections. However, this approach was capable of detecting a majority of nucleus-mitochondria OOIs and the quantitative differences did not interfere with our ability to analyze the effects of pharmacological compounds on these OOIs.

To the extent that molecules involved in nucleus-plastid and/or nucleus-mitochondria OOIs are affected by a given pharmacological compound, the prediction is that fewer OOIs would be observed between these organelles in cells treated with the corresponding compound. No significant change in the percentage of nucleus-mitochondria OOIs was observed in seedlings treated with latrunculin or oryzalin compared to mock-treated seedlings (Figure 7A). These results suggest that cytoskeletal proteins, such as actin and microtubules, do not participate in this type of OOI. In contrast, NEM significantly reduced the percentage of nucleus-mitochondria OOIs (0.3-fold, P ≤ 0.01), suggesting that molecules sensitive to NEM-mediated alkylation may be involved. Moreover, dual exposure to NEM and latrunculin or NEM and oryzalin did not induce a further reduction in the interaction rate beyond that of NEM alone, indicating the absence of a synergistic effect. It is impossible to predict which molecules might control the nucleus-mitochondria OOIs on the basis of this experiment because NEM immobilizes many other organelles and impacts fluid dynamics (cytoplasmic streaming) in plant cells [31]. A more extensive analysis, perhaps involving random mutagenesis of the Kaleidocell line, will be required to identify the molecules involved in nucleus-mitochondria OOIs. Analyses of nucleus-plastid OOIs in these same experiments did not reveal any changes in the presence of any of these pharmacological compounds (Figure 7B). Although these results do not allow us to form any conclusions about the molecular aspects of nucleus-plastid OOIs, they do suggest that molecules or mechanisms other than those involved in nucleus-mitochondria OOIs are responsible for regulating nucleus-plastid OOIs. The difference between nucleus-mitochondria and nucleus-plastid interactions also suggest that these interactions are active interactions and not passive interactions.

## Conclusion

We demonstrate that Kaleidocell is a useful tool to begin the exploration of OOIs. We have identified nucleus-chloroplast and nucleus-mitochondria interactions in root epidermal cells, and have demonstrated that NEM disrupts nucleus-mitochondria OOIs but not nucleus-plastid OOIs. These results suggest that different mechanisms are involved in regulating nucleus-mitochondria and nucleus-plastid OOIs. Kaleidocell should also be useful in experiments designed to determine if cell type, developmental stage and/or exogenous stimuli are capable of regulating or altering these interactions. The homozygous Kaleidocell line is available through Arabidopsis Biological Resource Center with the stock number CS16303.

## Methods

### Fluorescent proteins to tag mitochondria and nuclei

The mitochondrial localization signal sequence in cytochrome oxidase IV (CoxIV) of *Saccharomyces cerevisiae *was amplified from pCK CoxIV-GFP  [17] and fused to yellow fluorescent protein EYFP (Clontech, CA) by a three step PCR amplification. The primers used for amplifying the coxIV signal peptide were LM128 (5'-AgggATCCAAAATggTTTCACTACgTCAATCTATAAgA-3') and LM129 (5'-TTgCTCACCATgggTTTTTgCTgAAgCAgATATCT-3'). The primers used for amplifying EYFP from the pEYFP-C1 (Clontech) were LM130 (5'-AgCAAAAACCCATggTgAgCAAgggCgAggAgCTgT-3') and LM131 (5'-ACgAgCTCACTTgTACAgCTCgTCCATgCCgA-3'). The products of these two reactions were combined and a third amplification was performed using LM128 and LM131. The product of this amplification was cloned into pGEM-T (Promega) and sequenced. The coxIV::EYFP was subsequently removed from this vector using the BamHI and SacI sites that were included in primers LM128 and LM131, respectively. The coxIV-EYFP BamHI/SacI fragment was then inserted into pBI121 (Clontech) at BamHI/SacI, thereby replacing the β-glucuronidase gene of the pBI121 vector (Clontech), forming pBI121-CoxIV::EYFP. A HindIII and EcoRI digested fragment that encoding 35SP (cauliflower mosaic virus 35S promoter)-CoxIV::EYFP-NosT(nopalin synthase terminator) in pBI121-CoxIV::EYFP was inserted in HindIII and Sse8387I digested pKW102 vector via DNA adapter that encodes EcoRI and PstI. The HindIII and Sse8387I sites are located in an upstream of 35SP in pKW102 that expresses Gal4::ECFP::NLS, a chimeric gene encoding yeast nuclear localized protein Gal4, a nuclear localization signal from SV40 large T-antigen, and the cyan fluorescent protein ECFP (Clontech). The resulting vector, designated as pKW102-CY-MODC, hence expresses both CoxIV::EYFP and Gal4::ECFP::NLS from a single T-DNA.

### Fluorescent proteins to tag plastids

The EYFP region of pEYFP-C1 vector (Clontech) was removed with NheI/SacI and subcloned into pBluescript SK+ (Stratagene) at XbaI/SacI to form pBSK+-EYFP. This vector was subsequently digested with BamHI/SacI and the EYFP containing region was subcloned into pBI221 (Clontech) digested with the same restriction enzymes, forming the pBI221-EYFP vector. A 300 bp fragment, corresponding to the signal peptide of the Arabidopsis RecA protein that localized in chloroplasts (NM_106556)  was PCR amplified from an Arabidopsis cDNA library  using primers LM116 (5' CgggATCCATggATTCACAgCTAgTCTTgTC 3') and LM117 (5' gAAgATCTTCCATAgCTgCCTCTAAAgCCTT 3'). This fragment was cloned into the pGEM-T vector (Promega) and sequenced. The RecA signal peptide was subsequently removed from this vector using the restriction sites that were incorporated in the primers (BamHI/BglII) and subcloned into the BamHI site of pBI221-EYFP forming the pBI221-RecA::EYFP vector. The EYFP region of this vector was replaced with the red fluorescent protein DsRed2 (Clontech) at the AgeI/SacI sites, forming the pBI221-RecA::DsRed2 vector. The region containing RecA::DsRed2 was then transferred to pEL103  [21] at the BamHI/SacI sites, forming the pEL103-RecA::DsRed2 vector.

### Fluorescent protein to tag plasma membrane

HindIII digested 35SP-sGFP::CaM53-NosT fragment from pGFPnew/BDCaM53wt [20] was inserted in HindIII digested pEL103-RecA::DsRed2. sGFP::CaM53encodes a chimeric protein of soluble GFP (sGFP) and petunia calmodulin CaM53 that localized in plant plasma membrane. The vector was designated as pEL103-GR.

### Plant transformation

*Agrobacterium tumefaciens *strain GV3101/pMP90 was transformed with the vectors, pKW102-CY-MODC, pEL103-RecA::DsRed2, and pEL103-GR respectively. These *Agrobacterium *strains were then used to transform *Arabidopsis thaliana *(Col-0) as described previously [32].

### Plant screening and crossing

Zygosities of each transgenic line were determined by their sensitivity to kanamycin selection and by detection of the fluorescent proteins in their seedlings. Homozygous lines of transgenic *Arabidopsis *expressing Gal4-CFP and CoxIV-YFP were crossed to homozygous lines of transgenic *Arabidopsis *expressing Cam53BD-GFP. The resulting homozygous lines generated from the self pollination of this cross were then crossed to RecA-RFP homozygous plants to obtain the Kaleidocell lines. The line that most strongly expressed all transgenes was designated Kaleidocell.17. Kaleidocell.17 was self-pollinated in the following three generations. The line that expressed all transgenes in its all progenies was identified and designated Kaleidocell.17.1.7. The progenies of Kaleidocell.17.1.7 are referred to as Kaleidocell in this report.

### Plant growth conditions to establish the Kaleidocell line

The seeds were germinated on 0.75% agar plates containing a half-strength (1/2) MS salt (Sigma, CA) containing 100 mg/L of kanamycin after sterilizing in 50% diluted Crolox (Crolox, CA). The plates were kept in an environmental chamber in which a temperature was set at 21°C and a day cycle was set at 16 hours light and 8 hours dark. The plants selected were transplanted in soil and grown in the same chamber.

### Southern blotting

The Southern blotting was conducted based on the previously published method [15]. Hydroponically cultured homozygous Kaleidocell was used to extract the genomic DNA.

### Protoplast preparation

The protoplasts were prepared based on the previously published protocol [33]. About 50 seedlings of the one-week-old Kaleidocell line were used as starting material.

### Seedling preparation for the extended-hour observation

An one-weeks-old seedling was transferred to a chambered #1.5 cover glass (Lab-Tek II, Catalogue number 155360, Nalge Nunc International, IL) and 4 to 5 sheets of 11 × 21 cm water-soaked Kimwipes^® ^(Kimberky-Clark, GA) were folded and piled on the top of the seedling in the chamber so that a height of the piled Kimwipes were slightly (i.e., ~5 mm) higher than that of the chamber. A slide (75 mm × 22 mm, Catalogue number 2948, Corning, NY) was used as a lid for the chamber. The slide-covered chamber was then fixed on a microscope stage using labeling tape (1.9 mm width, Catalogue number 15-959, Fisher Scientific, PA) so that the slide could push the water-soaked Kimwipes towards the seedling being analyzed.

### Seedling preparation for the enclosing observation

Appoximately 10–20 Kaleidocell seeds were germinated on water-damp filter paper (Catalogue number 10001-055, Whatman, UK) in a 60 × 15 mm Petri dish (Catalogue number 08-757-13A, Fisher Scientific, NH) at room temperature. At 3 days the seedlings was transferred with 50 μl of distilled water to a micro slide (2984, Corning, NY) with a one-well spacer (20 mm diameter, 0.12 mm deep, S24736, Invitrogen, CA) attached. The seedlings were then sealed with a glass cover slip (No. 1 1/2, 25 mm, 24 × 40 mm, Corning, NY).

### Sample preparation for the pharmacological analysis

One-week-old seedlings were transferred to a 60 × 15 mm Petri dish filled with 10 ml of distilled water. Dimethylsulfoxide (DMSO) or water solution containing pharmacological compounds was added into the Petri dish and gently shaken for 1 h at about 15 rpm on a rotory shaker. The seedlings were first transferred to a new Petri dish filled with distilled water to rinse the compounds and then transferred to a slide (75 mm × 22 mm) with a 0.12 mm deep spacer (Catalogue number S24736, Invitrogen, CA). Distilled water was added in the space formed by the spacer and this was sealed with a #1.5 cover glass (Catalogue number 2640, Corning, NY).

Fifty millimolar N-Ethylmaleimide (NEM) (Sigma), 40 mM 2, 4-Dinitrophenol (DNP) (Sigma), 2.5 mM Latrunculin B (Sigma), and 100 mM Oryzalin (Sigma) were prepared in DMSO. One molar 2, 3-Butanedione monoxime (BDM) was prepared in distilled water.

### Microscopy: Laser scanning confocal microscopy

Carl Zeiss (Germany) LSM 510 Meta equipped with C-Apochromat 40× N.A. 1.2 water immersion lens was used to obtain the 3-D model of the root cells. Three lasers, 458 nm at 55% power, 488 nm at 6% power, and 543 nm at 72% power were used to excite CFP, GFP/YFP, and Red respectively. Emission passes 465 – 520 nm, 505 – 530 nm, and > 560 nm were used to detect signals excited with 458 nm, 488 nm, and 543 nm lasers respectively. Pinhole sizes were adjusted to 67 μm, 68 μm, and 73 μm for each laser channel. Stacks used to generate 3-D images consisted of 67 1024 × 1024 images with dimensions of 230.2 × 230.3 × 30.9 μm with a scan resolution of 0.22 × 0.22 × 0.47 μm. Scan speed was set to 1.60 μs/pixel and recorded as 8 bit data.

### Microscopy: Deconvolution microscopy

Delta Vision^® ^RT Restoration Imaging System (Applied Precision, WA) equipped with Olympus Plan Apo 60× N.A. 1.2 water immersion objective lens and CoolSNAP HQ CCD camera (Photometrics, AZ), was used to obtain time-lapse movies. Three layers of stacked images consisting of 1024 × 1024 pixel images were acquired with 2 × 2 Bining (with a resolution of 0.21 × 0.21 × 0.47 μm). Multi-band filter sets with single emitters and single exciters (Catalogue number 86000, Chroma, CT) were used to excite CFP, YFP and RFP sequentially (exciters: 436/10 nm, 492/18 nm, 580/20 nm, emitters 465/30 nm, 535/30 nm, 630/60 nm). Exposure times for CFP, YFP, and RFP were 0.4 sec, 0.4 sec, and 0.1 sec, respectively.

### Microscopy: Wide-field microscopy

A Leica DM RXA2 upright microscope equipped with HCX PL APO CS 40× N.A. 1.25 oil immersion (for protoplast imaging) and HCX PL APO 40× N.A. 0.75 air (for pharmacological analysis) objective lenses and SensiCam QE CCD camera (the Cooke, MI) was used. Four different filter sets (Catalogue number 31004V2, 31039, 31040, and 41335, Chroma, CT) were used to excite CFP, GFP, YFP, and RFP sequentially (exciters: 436/20 nm, 470/20 nm, 510/20 nm, 546/11 nm, emitters: 480/40 nm, 510/20 nm, 560/40 nm, 605/75 nm). Exposure time for each fluorescent protein was 1.0 sec.

### Image analysis: Creating a 3-D model, counting organelles, and tracking organelle movements

The stacked images acquired with the laser scanning confocal microscope were first deconvolved with the blind deconvolution algorithm (AutoQuant Imaging, NY). The output levels in each channel were manually adjusted to increase signal-to-background ratios. The movies of three-dimensional models of Kaleidocell were created using Imaris 5.7.0 (Bitplane AG, Switzerland).

### Image analysis: Creating time-lapse movies

The software softWoRx equipped with the Delta Vision^® ^RT Restoration Imaging System was used to capture sequential images for time-lapse movies. This image data was then used on Imaris 5.7.0 to track organelle movement.

### Image analysis: Protoplasts image and evaluating nucleus-mitochondria and nucleus-plastids interactions in the pharmacological analyses

The software ImageJ1.3.7 was used to compile the protoplast images and manually identify the organelle-organelle interactions. These images were manually manipulated to enhance the signal-to-background ratio.

## Competing interests

The authors declare that they have no competing interests.

## Authors' contributions

NK designed the experiments, constructed the DNA vectors, generated and molecularly analyzed the transgenic plants, and analyzed the 3-D images. DR conducted the pharmacological analysis and collected the homozygous line of the transgenic plant for donation. MLB analyzed a root meristem and cotyledon. MB established the homozygous line of the transgenic plant. YF analyzed the protoplasts. AM and LAM designed and constructed the RecA-RFP and CoxIV-YFP. All authors read and approved the final manuscript.

## Supplementary Material

Additional file 1 **A movie file of Figure **5. A Quick time movie file of Figure 5. A point of view is simultaneously changed in the virtual three-dimensional world. Click here for file

Additional file 2 **A movie file of Figure **6(A). A Quick time movie file of Figure 6(A). The duration is shown on the bottom left. Bar = 30 μm. Click here for file

Additional file 3 Enlarged image of Figure 6(B). Click here for file

Additional file 4 Enlarged image of Figure 6(C). Click here for file

Additional file 5 Enlarged image of Figure 6(D). Click here for file
